# Data on efficacy of umbelliferone on glycoconjugates and immunological marker in 7,12-dimethylbenz(a)anthracene induced oral carcinogenesis

**DOI:** 10.1016/j.dib.2017.09.035

**Published:** 2017-09-21

**Authors:** Annamalai Vijayalakshmi, Ganapathy Sindhu

**Affiliations:** Department of Biochemistry and Biotechnology, Faculty of Science, Annamalai University, Tamil Nadu, India

**Keywords:** Oral cancer, 7,12-dimethylbenz[a]anthracene, Glycoconjucates, Cytokeratin, Umbelliferone, Histopathology, Immunohistochemistry

## Abstract

Umbelliferone, a phenolic coumarin and dietary agent is believed to play a key role in pharmacological activities including anti-cancer and anti-oxidants effect in various *in vitro* and *in vivo* models. In present data on the pre-treatment of umbelliferone (30 mg/kg b.w.) for 16 weeks to 7,12-dimethylbenz(a)anthracene induced hamsters provides protection on cellular integrity by observing the status of cell surface glycoconjugates in the circulation and buccal mucosa and cytokeratin immunoexpression in the buccal mucosa of experimental animals. Data presented in this article brief that umbelliferone exhibits potent to clear cell surface abnormalities in buccal tissues and circulation during carcinogenesis and restored the expression of cytokeratin effect against 7,12-dimethylbenz(a)anthracene induced hamster buccal pouch carcinogenesis, which is attributes to its inhibitory role on glycoprotein synthesis or on the activity of the glycosyltransferase. In an article associates with this data set given the relevance to the research article entitled “Dose responsive efficacy of umbelliferone on lipid peroxidation, anti-oxidant, and xenobiotic metabolism in 7,12-dimethylbenz(a)anthracene-induced oral carcinogenesis” namely Vijayalakshmi and Sindhu, 2017 assessed 100% tumour formation in 7,12-dimethylbenz(a)anthracene treated hamsters and oral administration of umbelliferone at a dose of 30 mg/kg b.w to 7,12-dimethylbenz(a)anthracene treated hamsters prevents tumour incidence, restores the status of the biochemical markers in circulation and buccal mucosa and also dysregulation in the expression of molecular markers. Given the relevance to this article entitled “Berberine protects cellular integrity during 7,12-dimethylbenz[a]anthracene-induced oral carcinogenesis in golden Syrian hamsters” namely Sindhu and Manoharan 2010, which were based on spectrophotometry and florescence microscope analysis.

## Graphical abstract

**Figure:**
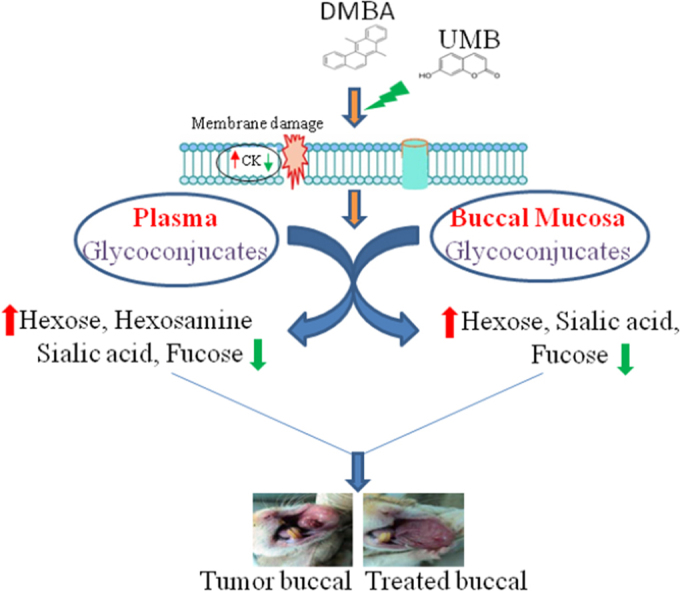

**Specifications Table**

**Table:** 

**Subject area**	*Oral cancer biology.*
**Most specific subject area**	*Oral biology, oral oncology, cell biology, cancer biology, histology and biochemistry.*
**Types of data**	*Figures*
**How the data were acquired**	*Spectrophotometer and fluorescence microscope (Nikon eclipses TS100).*
**Data format**	*Raw analyzed data.*
**Experimental factors**	*Biochemical analysis was determined in circulation and buccal tissue. Buccal tissue glycoproteins were assessed using spectrophotometry. Cytokeratin immunoexpression patterns were observed by using fluorescence microscope*
**Experimental features**	*Hamsters, 80–120* *g, procured from the National Institute of Nutrition, India and were maintained in the central animal house, in polypropylene cages, room temperature (27±2* *°C), humidity 55* *°C.*
**Data source location**	*Annamalai University, Department of Biochemistry and Biotechnology, Annamalainagar, India. Ramachandra University, Department of Oral pathology, Chennai.*
**Data accessibility**	*All data are provided with this article.*
**Related research articles**	*Vijayalakshmi and Sindhu**[1]**; Sindhu and Manoharan et al.**[2]**.*

**Value of the data**

● Data will be useful to understand the effect of umbelliferone on the biochemical and cellular changes in hamsters buccal mucosa.
● Umbelliferone mediated modulation of glucoproteins expression could be further investigated to find its role in signalling mechanism during neoplastic transformation.
● Data from this study using 7,12-dimethylbenz (a) anthracene induced hamster buccal pouch carcinogenesis will provide a standard model to test the chemotherapeutics and preventives.
● Present data described the pattern of glycoconjugate expression using biochemical, periodic acid sciffs staining and immunohistochemistry in the buccal mucosa and highlighted increased glycoconjugate expression, severe keratosis, hyperplasia, dysplasia and well differentiated squamous cell carcinoma, over expression of cytokeratin in tumor bearing hamsters.
● The data can be used to link the membrane stabilizing effects of umbelliferone on cell surface glycoconjugate expression oral carcinogenesis.

## 1 Data

Detection of glycoconjugates and cytokeratin expression could help to assess the cancer progression. The protective role of umbelliferone during oral carcinogenesis via the inhibitory role on an abnormal glycoprotein synthesis or regulatory glycosyl transferase activity which was observed through the restored expression of cytokeratins, in experimental animals. Histopathological and immunohistochemical findings of the present study indicates that membrane stabilising effect exerted by umbelliferone during 7,12-dimethylbenz(a)anthracene induced experimental carcinogenesis.

## 2 Experimental design, materials and methods

### 2.1 Experimental design

**Fig. 1:**
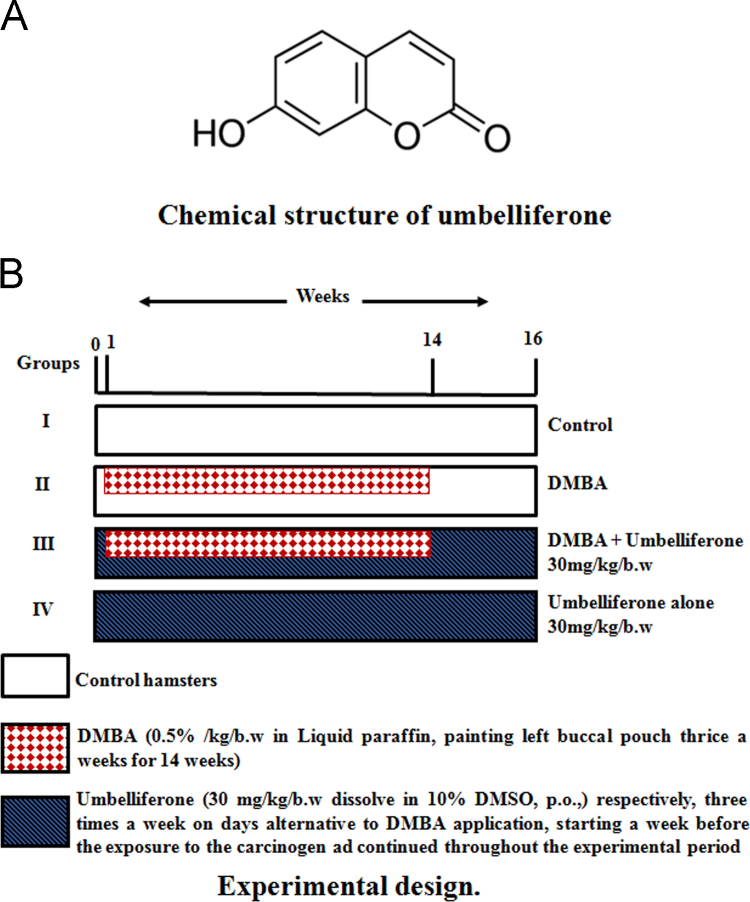
(A) Chemical structure of umbelliferone, (B) Experimental design.

A totally 24 hamsters were randomized into four groups (*n*=6). Tumour was induced in the left buccal pouch of hamster by topical application of 0.5% 7,12-dimethylbenz (a) anthracene in liquid paraffin thrice a week for 14 weeks. Fig. 1A shows umbelliferone (30 mg/kg b.w., dissolve in 10% dimethyl sulfoxide, *p.o*.,) three times a week on days alternative to 7,12-dimethylbenz(a)anthracene application, starting a week before the exposure to the carcinogen and continued throughout the experimental period. Fig. 1B was showed the experimental design. At 16th week, hamsters were euthanized by cervical dislocation, the buccal pouches were everted, and the degree of lesions were inspected to evaluate premalignant lesions and tumor development (Fig. 4A).

### 2.2 Biochemical studies

**Fig. 2:**
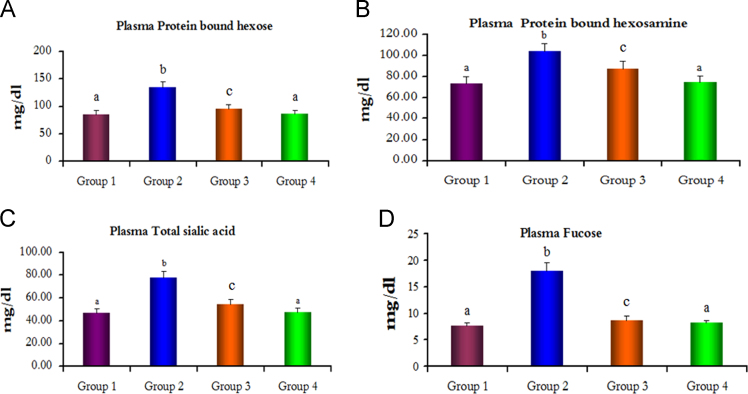
Protein bound hexose, protein bound hexosamine, total sialic acid and fucose levels in the plasma of control and experimental hamsters in each group. Bars are expressed as mean±SD for 6 animals in each group. (a–c) Values that do not share a common superscript letter between groups different significance, (a) no significantly different from group 1, (b) significantly different from group 1 at *p*<0.01, (c) significantly different from group 1 at *p*<0.05 (Duncan multiple range test).

**Fig. 3:**
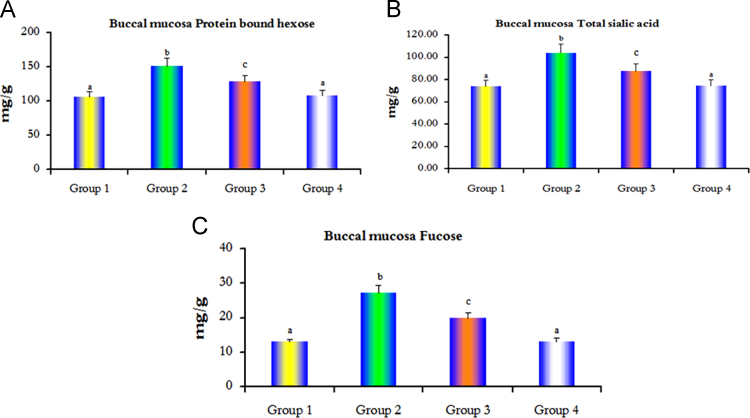
Protein bound hexose, total sialic acid and fucose levels in the buccal mucosa of control and experimental hamsters in each group. Bars are expressed as mean±SD for 6 animals in each group. (a–c) Values that do not share a common superscript letter between groups different significance, (a) no significantly different from group 1, (b) significantly different from group 1 at *p*<0.01, (c) significantly different from group 1 at *p*<0.05 (Duncan multiple range test).

The protein bound hexose and total sialic acid in plasma and defatted buccal tissue was estimated by the method of Niebes [3] and Dische [5] respectively. The protein bound hexosamine in plasma was estimated by the method of Wagner [4] Fig. 2, Fig. 3.

### 2.3 Histological analysis

**Fig. 4:**
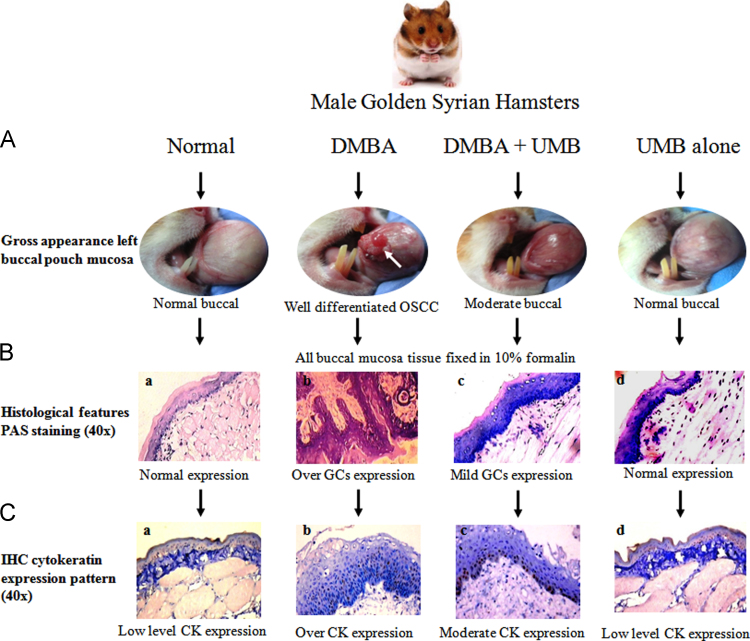
Experimental workflow for detection of glycoconjucates and cytokeratin expression on 7,12-dimethylbenz(a)anthracene induced hamsters buccal pouch carcinogenesis. (A) Photograph showing the gross appearance of oral squamous cell carcinoma (*n*=6) (40×). Exophytic well defined tumor mass in the hamsters painted with 7,12-dimethylbenz(a)anthracene (White arrow). (B**),** Histological features of glycoconjugates expression pattern observed in the buccal mucosa of control and experimental hamsters in each group (Periodic acid staining; Glycoconjugates, 40×). (a) showing normal glycoconjugates expression in buccal mucosa tissue, (b) showing over glycoconjugates expression in well differentiated squamous cell carcinoma with keratin pearls, (c) showing lowered glycoconjugates expression and mild dysplasia with hyperkeratosis, (d) showing the normal expression in umbelliferone alone treated hamsters. (C) Immunohistochemical staining of cytokeratin expression pattern in the buccal mucosa of control and experimental hamsters in each group (40×).

Tumour specimens were fixed in 10% (v/v) neutral formalin solution for 24 h and processed routinely by embedding in paraffin. Tissue serial sections (2–3 µm) were immersed with 0.1% periodic acid staining for 15 min, at 50 °C. The slides were immersed in schiff's reagent for 40 min, subsequently counterstained with hematoxylin, dehydrated in graded ethanol, cleared in xylene and examined under the light microscope (Nickon eclipse TS100, Japan) Fig. 4B.

### 2.4 Immunohistochemical analysis for cytokeratin expression

Immunohistochemical analysis for cytokeratin was performed according to the standard avidin-biotin complex method described in procedure programme of biotinylated rabit anti-mouse antibody and an avidin-biotin complex (Biogenesis, India). A previously known positive tumour tissues was used as a positive control for cytokeratin. The immunostaining grades were quantitatively analyzed by biological image analysis systems which consist of Nickon TS100 (Fig. 4C).

### 2.5 Statistical analysis

Data were expressed as mean±S.D. ANOVA and Student's *t*-test were used for determining differences between groups, and *P*≤0.05 was regarded as statistically significant.

## Ethical approval

The animal treatment and protocol employed was approved by the Institutional Animal Ethics Committee (Registration Number 160/1999/CPCSEA; Proposal No. 1092: dated 09.10.2014), Annamalai University. The animals were kept in compliance with the “Guide for the care and use of laboratory animals” and committee for the purpose of control and supervision on experimental animals.

## References

[1] Vijayalakshmi A., Sindhu G. (2017). Dose responsive efficacy of umbelliferone on lipid peroxidation, anti-oxidant, and xenobiotic metabolism in DMBA-induced oral carcinogenesis. Biomed. Pharmacother..

[2] Sindhu G., Manoharan S. (2010). Berberine protects cellular integrity during 7,12- dimethylbenz[a]anthracene- induced oral carcinogenesis in golden Syrian hamsters. Cell. Tissue Res..

[3] Niebes P., Berson I. (1973). Determination of enzymes and degradation product of glycosaminoglycan metabolism in the serum of health and various subjects. Bibl. Anat..

[4] Wagner W.D. (1979). A more sensitive assay discriminating galactosamine and glucosamine in mixture. Anal. Biochem..

[5] Dische L., Shettles L.B. (1948). Specific color reactions of methyl pentoses and spectrophotometric micromethod for their determination. J. Biol. Chem..

