# Supplementary material for: Data on efficacy of umbelliferone on glycoconjugates and immunological marker in 7,12-dimethylbenz(a)anthracene induced oral carcinogenesis
Source: Data Brief. 2017 Sep 21;15:216–21. doi: 10.1016/j.dib.2017.09.035 (PMC5633246; doi:10.1016/j.dib.2017.09.035)
Supplement: Supplementary file 1 — Transparency document [file mmc1.docx]

**Conflict of interest**

There is no conflict of interest
